# Social interaction and functional status among community-dwelling older adults in Japan: a longitudinal study during the coronavirus disease 2019 pandemic

**DOI:** 10.3389/fpubh.2025.1710300

**Published:** 2026-01-15

**Authors:** Jinrui Zhang, Meiling Qian, Shuanghong Li, Ruifeng Zhao, Dandan Jiao, Mingyu Cui, Yang Liu, Yuko Sawada, Akihiro Kakuda, Tokie Anme

**Affiliations:** 1 Empowerment Lab, Graduate School of Comprehensive Human Sciences, University of Tsukuba, Tsukuba, Japan; 2 Department of Nursing, The First Affiliated Hospital and College of Clinical Medicine of Henan University of Science and Technology, Luoyang, China; 3 Department of Nutrition and Food Hygiene, School of Public Health, Peking University, Beijing, China; 4 Key Laboratory of Digital-Intelligent Disease Surveillance and Health Governance, North Sichuan Medical College, Nanchong, China; 5 Department of Physical Therapy, Morinomiya University of Medical Sciences, Osaka, Japan; 6 Faculty of Medicine, University of Tsukuba, Tsukuba, Japan

**Keywords:** adaptability, aging, COVID-19, functional status, longitudinal study, social curiosity, social interaction

## Abstract

**Introduction:**

Previous studies have shown that social relationships positively contribute to functional status. However, studies comparing the influence under changing social circumstances are still limited. Consequently, this study investigated how social interaction influenced long-term functional status among older Japanese adults before and during the COVID-19 pandemic.

**Methods:**

A two-wave longitudinal cohort design was used to compare pre-pandemic (2017–2020) and post-pandemic (2020–2023) cohorts. Data were obtained from the Community Empowerment and Care study in T-Village, Aichi Prefecture, Japan. Social interaction was assessed using the Index of Social Interaction (ISI) and functional status was measured based on long-term care needs. Logistic regression analyzed predictors of functional health over 3 years, controlling for demographic and life-style covariates.

**Results:**

Social interaction declined during the pandemic. Higher ISI scores, particularly for social curiosity and adaptability, predicted better functional outcomes in both cohorts. Interaction with non-family members and willingness to use new technology were strong protective factors.

**Discussion:**

Promoting meaningful social engagement and behavioral adaptability may help preserve functional independence in aging populations, especially during periods of social disruption.

## 1 Introduction

Japan is undergoing an unparalleled demographic transition. By 2065, nearly 40% of its population will be aged 65 years or older, with a substantial proportion entering the “old-old” category (aged >75 years), in which functional decline becomes particularly pronounced (1). This demographic shift has led to increasing demands on Japan’s health and social care systems, with Long-Term Care Insurance (LTCI), introduced in 2000, emerging as a central mechanism for providing equitable support to older adults (2). Functional status, defined as the capacity to perform essential physical, cognitive, and social tasks of daily life, plays a pivotal role in determining LTCI eligibility and utilization (3). However, despite the availability of institutional and community-based services, a growing number of older adults experience significant decline, posing challenges to sustainable long-term care delivery (4).

A critical, yet sometimes underemphasized, determinant of functional health is social interaction. Social interaction encompasses the processes through which individuals initiate, maintain, and interpret relationships, ranging from everyday exchanges to deeper emotional bonds (5). Contemporary research increasingly highlights that social relationships are not merely supportive but constitutive of health in later life. Functional health does not deteriorate in a vacuum; rather, it unfolds within the social context of daily life. Evidence suggests that limited social engagement is associated with higher risks of physical limitations, cognitive impairment, and emotional distress, which together may accelerate functional decline (6, 7).

Theoretical framework. We interpret these associations using the biopsychosocial model and the social–ecological model: social relationships may shape functional health through intertwined behavioral and psychosocial pathways, while also reflecting influences operating across individual, interpersonal, and community contexts. A recent review on social isolation/loneliness and cognitive outcomes further supports the relevance of this multi-level, integrative perspective (8).

Several longitudinal studies have demonstrated that frequent and meaningful social contact is protective against adverse functional outcomes. For instance, older adults who engage in regular group-based activities, such as community exercise classes, volunteer groups, or senior clubs, show better maintenance of mobility, balance, and overall health than those who remain socially isolated (9). The mechanisms underlying these associations are both behavioral and psychosocial. Socially engaged individuals are more likely to remain physically active, adhere to healthy routines, and access informal caregiving networks when needed (10). Moreover, emotional support buffers against life stressors, such as bereavement, illness, or reduced income, that often precipitate psychological and physical decline (11).

Social interaction fosters cognitive stimulation, which has been shown to slow the progression of cognitive impairment, a key contributor to loss of independence in later life (12). Activities involving communication, planning, and shared experiences can enhance executive functions and memory, especially when interactions are frequent and emotionally meaningful. This is particularly important given that cognitive function is increasingly being incorporated into functional status assessments for LTCI eligibility in Japan (13). In this way, social engagement becomes both a preventive tool and an indirect policy lever for delaying LTCI certification and promoting healthy aging.

The quality of social relationships matters as much as the quantity. Low-quality or ambivalent relationships can exert negative effects, including increased stress and inflammation, which may negate the benefits of social contact (14). Therefore, it is not merely being connected but being meaningfully and positively connected that confers protective effects on functional health. Indicators such as perceived support, relational satisfaction, and reciprocity have emerged as key moderators in this relationship (15). Accordingly, we operationalize social relationships using the multidimensional ISI to capture these layered facets of social interaction in later life.

The COVID-19 pandemic has underscored the importance of social interaction for older adults. In response to the outbreak, Japan implemented nationwide social distancing policies and discouraged group activities and family visits, particularly in long-term care facilities. Although these measures were essential for infection control, they led to substantial reductions in face-to-face interaction among older adults (16). Evidence from Japan and other countries has shown that the resulting social isolation contributed to worsening physical activity levels, increased depressive symptoms, and declines in both cognitive and functional capacities (17, 18). In institutional settings, nearly half of older residents experienced a decline in activities of daily living within a few months of visitation bans (19).

Older adults face substantial barriers in transitioning to digital communication. Limited digital literacy, a lack of access to devices, and a preference for in-person contact hinder their ability to maintain social connections virtually (20). Even when digital tools are available, older adults report mixed experiences, with perceived lack of authenticity or usability reducing their emotional and psychological benefits (21). These findings suggest that social connectivity is not easily replaceable and highlight the multidimensionality of social interaction in aging populations.

Despite valuable insights from previous studies, significant gaps remain. Much of the existing literature relies on cross-sectional or short-term longitudinal designs, limiting the ability to draw causal inferences or assess long-term effects. Moreover, most studies employ narrow indicators of social engagement, such as contact frequency or living arrangements, which fail to capture the structural, functional, and emotional complexity of social life. A more nuanced understanding is therefore needed—one that incorporates multiple dimensions of social interaction and traces their evolution over time.

To address these limitations, the present study adopts a two-wave longitudinal cohort design within the same urban population in Japan to examine how changes in daily life influence functional aging. By comparing social interaction and functional status among older adults before and after the pandemic, the study provides a more comprehensive view of social dynamics over time and identifies the social and behavioral factors that help sustain independence under shifting circumstances. These findings offer critical evidence to inform the development of targeted, socially grounded interventions and policy strategies aimed at promoting healthy aging and reducing dependence on long-term care in rapidly aging societies such as Japan.

Based on the existing literature regarding the impact of social distancing measures, this study aims to examine the longitudinal changes in social interaction and their association with functional status among Japanese older adults. We formulated two primary hypotheses: (1) differences exist in social interactions between the pre- and post-COVID-19 outbreak periods; and (2) older adults with higher levels of social interaction demonstrate better functional status compared to those with lower levels of social interaction.

## 2 Methods

### 2.1 Study design and data source

This longitudinal study followed community-dwelling older adults from 2017 to 2023, divided into two waves. Wave 1 covered 2017–2020 and Wave 2 covered 2020–2023. The two waves reflect different stages of change: one group experienced the transition into the pandemic, whereas the other experienced the shift toward recovery. This approach made it possible to observe how different conditions were linked to resilience and identify factors that appeared important in supporting function under each context.

Data were obtained from the “Community Empowerment and Care for Well-being and Healthy Longevity: Evidence from Cohort Study” (CEC), which was initiated in 1991 to identify determinants of health and longevity in suburban communities (22). The survey was conducted in T-Village, Aichi Prefecture, Japan, a suburban area with approximately 4,800 residents. The area had an aging rate of 28.2% in 2017 and 28.5% in 2020, making it a representative setting for studying the rural–urban transition.

In each wave, residents aged ≥65 years were invited to participate. Questionnaires included demographic information, health behaviors, chronic conditions, and social interaction. Local staff and volunteers distributed and collected surveys by visiting households, aiding those unable to complete the forms independently.

### 2.2 Participants and procedure

The inclusion criteria were community-dwelling individuals aged 65 years and older. Participants were excluded if they required long-term care or had missing data on long-term care status. The participant flow for each wave is detailed below:

Wave 1 (2017–2020): From an initial sample of 1,087 older adults, 768 participants were retained at baseline after excluding individuals who required care (*n* = 181) or had missing data (*n* = 138). By the 2020 follow-up, 449 participants remained, with 319 lost owing to death, hospitalization, or relocation.

Wave 2 (2020–2023): From an initial sample of 688 older adults, 554 participants were retained at baseline after excluding individuals who required care (*n* = 88) or had missing data (*n* = 46). By the 2023 follow-up, 364 participants remained, with 190 lost to follow-up.

Records were linked using unique participant IDs. We identified 245 participants who were observed in both 2017 and 2020.

### 2.3 Measures

#### 2.3.1 Social interaction

Social interaction was used to assess using the ISI, which demonstrated acceptable internal consistency (Cronbach’s *α* = 0.78).

The ISI encompasses five subscales across 18 items (23), each targeting a distinct facet of social interaction. The Independence subscale (4 items) assesses personal drive for purposeful living, proactive life engagement, maintenance of a regular daily routine, and health-oriented motivation. The Social Curiosity subscale (5 items) explores behaviors such as reading newspapers and books, experimenting with new technologies, pursuing hobbies, and perceiving oneself as socially relevant. The Interaction subscale (3 items) measures the extent of communication with family members, non-family acquaintances, and general interpersonal engagement beyond the family unit. The Social Participation subscale (4 items) evaluates involvement in community-based or neighborhood activities, group affiliations, TV consumption, and active societal contribution. The Feeling of Safety subscale (2 items) gages whether individuals have access to someone they can consult or rely on in times of emergency or need.

Responses of “always/often/sometimes” were coded as 1, and “rarely” as 0. The total ISI score ranged from 0 to 18, with higher scores indicating stronger social ties (24). The ISI total score and subscale scores were treated as continuous variables, whereas individual ISI items were treated as dichotomous variables.

#### 2.3.2 Functional status

Functional status was assessed using a question from the Survey of Needs in the Sphere of Daily Life developed by Japan’s Ministry of Health, Labor and Welfare (25). Participants were asked: “Do you need care or assistance from someone in your daily life?” Response options were: “No, I do not need care/assistance”; “I need some care/assistance, but I am not currently receiving it”; and “I am currently receiving some care/assistance (including informal care provided by family members without long-term care certification)”. Respondents answering “no need” for support were considered as high-functioning, whereas those requiring care or support were considered as low-functioning.

Guided by the disablement process framework, we operationalized functional status as self-reported need for care/assistance in daily life, reflecting a disability-related dependency state rather than a multi-item scale (26). Because long-term care and care dependency are widely recognized concepts in Japan, this item is expected to capture respondents’ perceived assistance needs in daily life and therefore serves as a pragmatic proxy indicator of care dependency/functional limitation (27).

#### 2.3.3 Covariates

Several self-reported covariates were included to control for potential confounding variables. These included age, sex, body mass index (BMI), living status, subjective physical activity, alcohol consumption, smoking habits, and the presence of chronic health conditions. These covariates were included simultaneously in all multivariable logistic regression models.

### 2.4 Statistical analysis

All statistical analyses were performed using IBM SPSS Statistics, Version 29 (Armonk, NY, United States). First, descriptive statistics were used to analyze categorical variables. Additionally, inferential comparisons between 2017 and 2020 were conducted using Wilcoxon signed-rank tests and McNemar tests. Sample composition was additionally examined by comparing overlapping participants with those observed in only 1 year within each survey year using Mann–Whitney U tests and chi-square/Fisher’s exact tests.

Second, we used multivariable logistic regression to examine associations between social relationships and low functional status 3 years later in each wave. ISI total score, each subscale score, and each item were modeled separately (i.e., one ISI predictor per model) to avoid collinearity. All models were adjusted for the same 8 covariates. A significance level of *p* < 0.05 was established for all analyses. The generic model formula was:


$$\text{logit}\{P(\mathrm{low}\;\text{function}=1)\}=\beta_{0}+\beta_{1}X_{\mathrm{ISI}}+\sum_{k}\beta_{k}C_{k}$$

Model stability was assessed using events per predictor (EPV); EPV values for Wave 1 and Wave 2 were 2.89 and 4.44.

### 2.5 Ethical consideration

This study was approved by the Institutional Review Board of the University of Tsukuba (approval no. 1331–7). The municipality supplied the data under formal agreements, and all records were anonymized. In accordance with ethical procedures, residents were informed of the study and given the opportunity to decline participation.

## 3 Results

Table 1 shows the descriptive statistics of demographic characteristics of the participants in 2017 and 2020. In 2020, the proportion of participants aged ≥75 years increased to 33.0% compared with 27.6% in 2017, whereas other demographic and lifestyle variables, including sex, BMI, living status, exercise, smoking, drinking, and chronic disease prevalence, were broadly similar across the 2 years. Paired comparisons among overlapping participants are presented in Table 2.

**Table 1:** Demographic characteristics in 2017 and 2020.

Items	Categories	2017		2020	
		*n*	%	*n*	%
Age, years	65–74	325	72.4	244	67.0
	≥75	124	27.6	120	33.0
Sex	Male	201	44.8	158	43.4
	Female	248	55.2	206	56.6
BMI	Normal	435	94.7	338	92.9
	Not normal	24	5.3	26	7.1
Living status	Else	339	75.5	257	70.6
	Alone	110	24.5	107	29.4
Exercise	Active	297	66.1	241	66.2
	Inactive	152	33.9	123	33.8
Smoking	Never	293	65.3	235	64.6
	Smoker	156	34.7	129	35.4
Drinking	Non-daily	351	78.2	281	77.2
	Daily	98	21.8	83	22.8
Chronic diseases	0	75	16.7	68	18.7
	≥ 1	374	83.3	296	81.3
Social interaction (mean ± SD)					
Total score		16.48 ± 1.88		16.05 ± 2.32	
Independence		3.94 ± 0.31		3.82 ± 0.74	
Social curiosity		4.45 ± 0.91		4.35 ± 0.93	
Interaction		2.90 ± 0.34		2.79 ± 0.50	
Participation in the society		3.65 ± 0.57		3.56 ± 0.64	
Feeling of safety		1.90 ± 0.39		1.85 ± 0.45	

BMI: body mass index.

**Table 2:** Paired comparisons of ISI total and domain scores between 2017 and 2020 among overlapping participants (*n* = 245).

Items	2017 (mean ± SD)	2020 (mean ± SD)	*W+*	*W−*	*r_rb_*	*p-*value
ISI total score	16.61 ± 1.82	16.07 ± 2.17	5101.0	10299.0	−0.338	<0.001
Social curiosity	4.45 ± 0.92	4.30 ± 0.94	2119.5	4096.5	−0.318	0.002
Interaction	2.91 ± 0.35	2.77 ± 0.51	300.0	1131.0	−0.581	<0.001
Feeling of safety	1.91 ± 0.39	1.87 ± 0.42	279.0	424.0	−0.206	0.249

Values are mean ± SD. W + and W − are sums of positive and negative signed ranks, respectively; r_rb_ is the rank-biserial correlation. *p* values were obtained using Wilcoxon signed-rank tests (two-sided). ISI, Index of Social Interaction; SD, standard deviation.

Table 2 shows that within overlapping participants (*n* = 245), paired analyses showed a decrease in ISI total score from 2017 to 2020 (16.61 ± 1.82 to 16.07 ± 2.17; *p* < 0.001). Domain-level comparisons indicated significant decreases in social curiosity (4.45 ± 0.92 to 4.30 ± 0.94; *p* = 0.002) and interaction (2.91 ± 0.35 vs. 2.77 ± 0.51; p < 0.001), whereas the change in feeling of safety was not statistically significant (1.91 ± 0.39 to 1.87 ± 0.42; *p* = 0.249).

Table 3 presents sample composition comparisons and suggested potential follow-up selection, as overlapping participants had slightly higher ISI total scores in 2017 than those observed only in 2017 (16.61 ± 1.82 vs. 16.30 ± 1.95, *p* = 0.042). Moreover, in 2020, overlapping participants showed a higher proportion aged ≥75 years than those observed only in 2020 (37.1% vs. 24.4%), although this difference was not statistically significant (*p* = 0.091).

**Table 3:** Sample composition comparisons between overlapping and single-year participants.

Year	Indicator	Overlapping participants	Single-year participants	Test statistic	*p-*value
2017	ISI total score (mean ± SD)	16.61 ± 1.82 (*n* = 245)	16.30 ± 1.95 (*n* = 204)	26,850	0.042
2020	Age ≥75 years, n (%)	91 (37.1%) (*n* = 245)	29 (24.4%) (*n* = 119)	2.85	0.091

Values are mean ± SD or *n* (%). For 2017, overlapping participants were compared with participants observed only in 2017; for 2020, overlapping participants were compared with participants observed only in 2020. P values were obtained using Mann–Whitney U tests (continuous variables) and chi-square tests or Fisher’s exact tests (categorical variables), as appropriate (two-sided). ISI, Index of Social Interaction; SD, standard deviation.

Table 4 compares associations between demographic variables, ISI scores, and functional outcomes in both waves. In Wave 1 (2017–2020), participants aged 75 years or older were more likely to experience functional decline (*p* = 0.010), whereas higher ISI total and social curiosity scores were associated with better functional preservation. In Wave 2 (2020–2023), the same pattern was observed; older age (*p* = 0.049) increased the risk of decline, whereas higher ISI scores and curiosity remained protective.

**Table 4:** Associations between demographic characteristics, total ISI score, and domains and functional status across two waves.

Items	Wave 1 (*n* = 449)			Wave 2 (*n* = 364)		
	High group	Low group	*p-*value	High group	Low group	*p-*value
Age, *n* (%)						
65–74	312 (73.8)	13 (50.0)	0.010	218 (67.3)	14 (35.0)	0.049
≥75	111 (26.2)	13 (50.0)		106 (32.7)	26 (65.0)	
ISI score, mean ± SD	16.53 ± 1.84	15.73 ± 2.20		16.05 ± 2.32	16.08 ± 2.37	<0.001
Social curiosity, mean ± SD	4.48 ± 0.75	4.33 ± 0.95		4.45 ± 0.91	3.96 ± 1.40	0.001

ISI, Index of Social Interaction.

Table 5 presents item-level associations across waves. In Wave 1, “motivation to live” (*p* = 0.019) and “trying to use new equipment” (*p* = 0.013) were significantly related to functional preservation. In Wave 2, however, the strongest predictors shifted: “interaction with non-family persons” (*p* = 0.002) and “trying to use new equipment” (*p* = 0.010) were significant, whereas “motivation to live” was no longer significant. This shift indicates that internal motivation was more relevant before the pandemic, whereas external social connections became more important during the pandemic.

**Table 5:** Associations between ISI individual items and functional status across two waves.

Items	Wave 1 (*n* = 449)			Wave 2 (*n* = 364)		
	High group	Low group	*p-*value	High group	Low group	*p-*value
Interaction with nonfamily persons						
Yes	401 (94.8)	25 (96.2)	0.042	290 (89.5)	35 (87.5)	0.002
No	22 (5.2)	2 (3.8)		34 (10.5)	5 (12.5)	
Try to use new equipment						
Yes	360 (85.1)	18 (69.2)	0.013	240 (74.1)	34 (95.0)	0.010
No	63 (14.9)	8 (30.8)		84 (25.9)	6 (15.0)	
Motivation to live						
Yes	411 (97.2)	23 (88.5)	0.019	306 (94.4)	38 (95.0)	0.052
No	12 (2.8)	3 (11.5)		18 (5.6)	2 (5.0)	

ISI, Index of Social Interaction.

Table 6 summarizes logistic regression results for both waves. In Wave 1, higher ISI scores (OR = 2.69, 95% CI = 1.13–6.44, *p* = 0.025), social curiosity (OR = 2.70, 95% CI = 1.18–6.18, *p* = 0.018), trying to use new equipment (OR = 2.37, 95% CI = 1.01–5.23, *p* = 0.047), and motivation to live (OR = 1.30, 95% CI = 1.28–6.20, *p* = 0.013) predicted functional preservation. In Wave 2, ISI scores (OR = 3.34, 95% CI = 1.61–6.93, *p* = 0.001), social curiosity (OR = 2.79, 95% CI = 1.23–6.36, *p* = 0.014), interaction with non-family persons (OR = 1.55, 95% CI = 1.36–1.82, *p* = 0.003), and trying to use new equipment (OR = 2.82, 95% CI = 1.24–6.43, *p* = 0.024) were significant predictors.

**Table 6:** Associations between social interaction and functional status 3 years later.

Items	Wave 1 (*n* = 449)			Wave 2 (*n* = 364)		
	OR	95% CI	*p*-value	OR	95% CI	*p*-value
ISI score	2.69	1.13–6.44	0.025	3.34	1.61–6.93	0.001
Social curiosity	2.70	1.18–6.18	0.018	2.79	1.23–6.36	0.014
Interaction with nonfamily persons	0.57	0.18–1.74	0.322	1.55	1.36–1.82	0.003
Try to use new equipment	2.37	1.01–5.23	0.047	2.82	1.24–6.43	0.024
Motivation to live	1.30	1.28–6.20	0.013	0.30	0.39–3.75	0.737

ISI, Index of Social Interaction; OR, Odds ratio; CI, Confidence interval.

## 4 Discussion

The research findings supported the study hypotheses. Specifically, the study found a decrease in social interaction during the pandemic, while individuals who maintained a higher level of social interaction exhibited a better functional status 3 years later. These findings not only confirm the association between social interaction and healthy aging but also show the consistency of social curiosity and adaptability (measured by willingness to use new equipment) associations with functional maintenance across both waves. Moreover, the change from “motivation to live” before the pandemic to “non-family interaction” during the pandemic suggests that the aspects of social engagement that were strongly associated with functional health differed between the two waves.

This study examined how the COVID-19 pandemic affected social interaction among older Japanese adults and its long-term impact on functional status. A decline in social interaction from 2017 to 2020 was observed, aligning with national and international evidence that pandemic restrictions significantly reduced social opportunities for older adults and increased risks of isolation and loneliness (28, 29). Although Japan’s measures were less stringent than Western lockdowns, they still led to a decrease in community activity, with lingering effects on social participation and physical health (30, 31). Crucially, individuals who maintained active social engagement, through hobbies, conversation, and intellectual activity, were significantly more likely to preserve functional abilities over the subsequent 3 years. These findings are supported by studies showing that consistent social involvement buffers against cognitive and physical decline, even in populations with chronic illness (32, 33). Social frailty, marked by reduced participation and perceived social value, has also been tied to worse functional outcomes, with cognitive status mediating these effects (34).

Our findings can be interpreted in light of the proposed theoretical framework. From a biopsychosocial perspective, the observed associations between social relationships (ISI) and functional status are consistent with pathways involving sustained engagement in daily activities and psychosocial resources (e.g., emotional support and stress buffering). From a social–ecological perspective, the pattern across ISI domains suggests that functional health may be shaped not only by individual factors but also by interpersonal ties and opportunities for community participation. This interpretation aligns with recent evidence emphasizing multi-level approaches when addressing social isolation/loneliness and downstream cognitive and functional risks (8).

International research confirms that regular social engagement supports well-being, reduces inactivity, and mitigates depression across diverse cultures (35). One important explanation is that social engagement fosters healthier routines, emotional support, and a sense of meaning, factors that collectively promote mental and physical resilience (35, 36). Community involvement, especially through non-family ties and group activities, is particularly protective for individuals at risk of social isolation (37). The study also highlights the special role of social curiosity, the intrinsic motivation to learn about others, which was linked to better adaptability and functional health. Older adults with high curiosity were more likely to adopt new technologies and engage with diverse social contexts, supporting prior work connecting curiosity to openness, learning, and functional competence (38, 39). However, effect sizes in previous studies have been modest, suggesting that curiosity alone does not fully explain health trajectories and must be considered alongside social, cognitive, and motivational variables (40, 41).

A shift in predictors was also observed between 2017 and 2020; before the pandemic, internal motivation (i.e., “motivation to live”) was the key factor in maintaining function, but during the pandemic, external social ties, particularly with non-family members, became more critical. This suggests that, under disrupted conditions, access to supportive networks plays a heightened role in preserving health (42, 43). A consistent predictor across both time points was behavioral adaptability, measured by willingness to use new equipment. Technology adoption reflects a broader readiness to adapt and those who embrace communication tools or assistive devices are more likely to stay connected and maintain autonomy (44, 45). Despite common barriers such as digital literacy and access, interventions such as community-based training have proven effective in supporting technology use and bridging the digital divide (46, 47).

These results point to key policy and clinical implications. Public health programs should go beyond a general encouragement for social participation and instead focus on enhancing behavioral adaptability, digital competence, and non-kin relationships. Clinically, social engagement indicators, such as the frequency of contact and technology use, should be incorporated into geriatric assessments to identify individuals at risk of functional decline. This research contributes novel insights by using longitudinal data to track the effects of this pandemic, identifying social curiosity as a predictor of functional health, and underscoring the importance of adaptability in changing environments.

Limitations of this study should be noted. First, the paired analyses were based on participants with repeated observations, and sample composition comparisons suggested possible follow-up selection, therefore, generalization beyond individuals with repeated observations should be made with caution. Moreover, a substantial proportion of participants were lost to follow-up, which may have introduced attrition bias and further limited generalizability. Finally, this study relied on self-report data, which may limit measurement precision and sensitivity, in particular, functional status was assessed using a single self-reported item on need for care/assistance, which may not fully capture multidimensional functioning and may introduce some misclassification. Future studies should use diverse samples, mixed methods, and more granular health measures to promote social interaction and adaptability among older adults.

## 5 Conclusion

Older Japanese adults with higher social interaction during both the pre- and post-pandemic were more likely to maintain functional health. Adaptability, such as using new communication tools, consistently supported well-being. The findings highlight the need for targeted interventions to promote social connection in aging populations.

## Data Availability

The data analyzed in this study is subject to the following licenses/restrictions: The datasets generated and/or analyzed during the current study are available from the corresponding author upon reasonable request and with approval from the municipal government, owing to privacy restrictions. Requests to access these datasets should be directed to Tokie Anme, tokieanme@gmail.com.

## References

[1] Liu Y Kobayashi S Karako K Song P Tang W. The latest policies, practices, and hotspots in research in conjunction with the aging of Japan’s population. Biosci Trends. (2024) 18:219–23. doi: 10.5582/bst.2024.01150, 38866487

[2] Olivares-Tirado P Tamiya N Kashiwagi M. Effect of in-home and community-based services on the functional status of elderly in the long-term care insurance system in Japan. BMC Health Serv Res. (2012) 12:239–9. doi: 10.1186/1472-6963-12-239, 22863362 PMC3505459

[3] Lu Y Sato K Nagai M Miyatake H Kondo K Kondo N. Machine learning–based prediction of functional disability: a cohort study of Japanese older adults in 2013-2019. J Gen Intern Med. (2023) 38:2486–93. doi: 10.1007/s11606-023-08215-2, 37127751 PMC10465410

[4] Watanabe R Tsuji T Ide K Noguchi T Yasuoka M Kamiji K . Predictive validity of the modified Kihon checklist for the incidence of functional disability among older people: a 3-year cohort study from the JAGES. Geriatr Gerontol Int. (2022) 22:667–74. doi: 10.1111/ggi.14439, 35843630 PMC9540013

[5] Hoppler SS Segerer R Nikitin J. The six components of social interactions: actor, partner, relation, activities, context, and evaluation. Front Psychol. (2022) 12:743074. doi: 10.3389/fpsyg.2021.743074, 35082713 PMC8784599

[6] Fan Z Lv X Tu L Zhang M Yu X Wang H. Reduced social activities and networks, but not social support, are associated with cognitive decline among older Chinese adults: a prospective study. Soc Sci Med. (2021) 289:114423. doi: 10.1016/j.socscimed.2021.114423, 34597879

[7] Yang G D’Arcy C. Physical activity and social support mediate the relationship between chronic diseases and positive mental health in a national sample of community-dwelling Canadians 65+: a structural equation analysis. J Affect Disord. (2022) 298:142–50. doi: 10.1016/j.jad.2021.10.055, 34728294

[8] Lazzari C Rabottini M. COVID-19, loneliness, social isolation and risk of dementia in older people: a systematic review and meta-analysis of the relevant literature. Int J Psychiatry Clin Pract. (2022) 26:196–207. doi: 10.1080/13651501.2021.1959616, 34369248

[9] Zimmer C McDonough M Hewson J Toohey A Din C Crocker P . Social support among older adults in group physical activity programs. J Appl Sport Psychol. (2023) 35:658–79. doi: 10.1080/10413200.2022.2055223

[10] Steinhoff P Reiner A. Functional social support and physical activity in community-dwelling older adults: a scoping review. Eur J Pub Health. (2023) 33:1417. doi: 10.1093/eurpub/ckad160.1417 PMC11103817 38769563

[11] Unger J Johnson C Marks G. Functional decline in the elderly: evidence for direct and stress-buffering protective effects of social interactions and physical activity. Ann Behav Med. (1997) 19:152–60. doi: 10.1007/BF02883332, 9603690

[12] Jin Y Si H Qiao X Tian X Liu X Xue Q . Relationship between frailty and depression among community-dwelling older adults: the mediating and moderating role of social support. Gerontologist. (2020) 60:1466–75. doi: 10.1093/geront/gnaa072, 32556208

[13] Takahashi S Yonekura Y Takanashi N Tanno K. Risk factors of long-term care insurance certification in Japan: a scoping review. Int J Environ Res Public Health. (2022) 19:2162. doi: 10.3390/ijerph19042162, 35206350 PMC8872097

[14] Mogic L Rutter E Tyas S Maxwell C O’Connell M Oremus M. Functional social support and cognitive function in middle- and older-aged adults: a systematic review of cross-sectional and cohort studies. Syst Rev. (2023) 12:86. doi: 10.1186/s13643-023-02251-z, 37211612 PMC10200705

[15] Shankar A McMunn A Demakakos P Hamer M Steptoe A. Social isolation and loneliness: prospective associations with functional status in older adults. Health Psychol. (2017) 36:179–87. doi: 10.1037/hea0000437, 27786518

[16] Noguchi T Kubo Y Hayashi T Tomiyama N Ochi A Hayashi H. Social isolation and self-reported cognitive decline among older adults in Japan: a longitudinal study in the COVID-19 pandemic. J Am Med Dir Assoc. (2021) 22:1352–6. doi: 10.1016/j.jamda.2021.05.015, 34107288

[17] Abe T Nofuji Y Seino S Hata T Narita M Yokoyama Y . Physical, social, and dietary behavioral changes during the COVID-19 crisis and their effects on functional capacity in older adults. Arch Gerontol Geriatr. (2022) 101:104708. doi: 10.1016/j.archger.2022.104708, 35489311 PMC9022396

[18] Manca R De Marco M Venneri A. The impact of COVID-19 infection and enforced prolonged social isolation on neuropsychiatric symptoms in older adults with and without dementia: a review. Front Psych. (2020) 11:585540. doi: 10.3389/fpsyt.2020.585540, 33192732 PMC7649825

[19] Kingsbury L Hong W. A multi-brain framework for social interaction. Trends Neurosci. (2020) 43:651–66. doi: 10.1016/j.tins.2020.06.008, 32709376 PMC7484406

[20] Seckman C. The impact of COVID-19 on the psychosocial well-being of older adults: a literature review. J Nurs Scholarsh. (2023) 55:97–111. doi: 10.1111/jnu.12824, 36218196 PMC9874600

[21] Derrer-Merk E Reyes-Rodríguez M Soulsby L Roper L Bennett K. Older adults’ experiences during the COVID-19 pandemic: a qualitative systematic literature review. BMC Geriatr. (2023) 23:580. doi: 10.1186/s12877-023-04282-6, 37730571 PMC10510218

[22] Anme T. *Community Empowerment and Care for Well-being and Healthy Longevity: Evidence from Cohort Study (CEC)*. (2015). Available online at: http://plaza.umin.ac.jp/~empower/cec/en/ (Accessed September 21, 2025).

[23] Anme T Takayama T. Health and social welfare study on the evaluation of social relationships: the development and validity of social relationships evaluation for community-dwelling older adults. Jpn J Soc Welf. (1995) 36:59–73. doi: 10.24469/jssw.36.2_59

[24] Anme T. Evaluation of environmental stimulation and its relation to physical deterioration in the elderly after 3 years – a health – social longitudinal study. Nihon Koshu Eisei Zasshi. (1997) 44:159–66. 9175406

[25] Ministry of Health, Labour and Welfare (Japan). *Summary of insured long-term care service report*. Available online at: Available online at: https://www.mhlw.go.jp/content/12301000/000972604.pdf (Accessed September 21, 2025).

[26] Verbrugge LM Jette AM. The disablement process. Soc Sci Med. (1994) 38:1–14. doi: 10.1016/0277-9536(94)90294-1, 8146699

[27] Watanabe K Tanaka E Watanabe T Chen W Wu B Ito S . Association between the older adults’ social relationships and functional status in Japan. Geriatr Gerontol Int. (2017) 17:1522–6. doi: 10.1111/ggi.12909, 27726293

[28] Takashima R Onishi R Saeki K Hirano M. Perception of COVID-19 restrictions on daily life among Japanese older adults: a qualitative focus group study. Healthcare (Basel). (2020) 8:450. doi: 10.3390/healthcare8040450, 33139662 PMC7711814

[29] Ito T Hirata-Mogi S Watanabe T Sugiyama T Jin X Kobayashi S . Change of use in community services among disabled older adults during COVID-19 in Japan. Int J Environ Res Public Health. (2021) 18:1148. doi: 10.3390/ijerph18031148, 33525441 PMC7908432

[30] Oguma Y Doihara N Saito Y Ito T Tajima T. Changes in physical activity and health indicators in the Covid-19 disasters: results of a mail questionnaire survey of community-based group exercise elderly participants in Fujisawa, Japan. Med Sci Sports Exerc. (2021) 53:193. doi: 10.1249/01.mss.0000761308.44385.12

[31] Jiao D Zhu Y Zhu Z Li X Zhang J Cui M . The trajectory of functional status among older adults with chronic diseases and the association with social relationships. Front Public Health. (2025) 13:1492489. doi: 10.3389/fpubh.2025.1492489, 40260172 PMC12009918

[32] Cui M Jiao D Miura K Liu Y Li X Zhu Z . Social frailty and functional status in Japanese older adults: the mediating role of subjective cognitive function. J Am Med Dir Assoc. (2024) 25:104971. doi: 10.1016/j.jamda.2024.02.009, 38537667

[33] Oh S Cho E Kang B. Social engagement and cognitive function among middle-aged and older adults: gender-specific findings from the Korean longitudinal study of aging (2008-2018). Sci Rep. (2021) 11:15876. doi: 10.1038/s41598-021-95438-0, 34354162 PMC8342413

[34] Luo M Ding D Bauman A Negin J Phongsavan P. Social engagement pattern, health behaviors and subjective well-being of older adults: an international perspective using WHO-SAGE survey data. BMC Public Health. (2020) 20:99. doi: 10.1186/s12889-019-7841-7, 31973695 PMC6979381

[35] Yen H Chi M Huang H. Social engagement for mental health: an international survey of older populations. Int Nurs Rev. (2022) 69:359–68. doi: 10.1111/inr.12737, 34874057

[36] Wang Y Chen Z Zhou C. Social engagement and physical frailty in later life: does marital status matter? BMC Geriatr. (2021) 21:248. doi: 10.1186/s12877-021-02194-x, 33858354 PMC8047563

[37] Lv R Yang L Li J Wei X Ren Y Wang W . Relationship between social participation and life satisfaction in community-dwelling older adults: multiple mediating roles of depression and cognitive function. Arch Gerontol Geriatr. (2024) 117:105233. doi: 10.1016/j.archger.2023.105233, 37956587

[38] Kashdan T Disabato D Goodman F McKnight P. The five-dimensional curiosity scale revised (5DCR): briefer subscales while separating overt and covert social curiosity. Pers Individ Dif. (2020) 157:109836. doi: 10.1016/j.paid.2020.109836

[39] Kashdan T McKnight P Kelso K Craig L Guenoun B Naughton C. Multiple dimensions of workplace curiosity: evidence of generalizability in nine countries. Pers Individ Dif. (2025) 236:3011. doi: 10.1016/j.paid.2024.113011

[40] Halverson T Orleans-Pobee M Merritt C Sheeran P Fett A Penn D. Pathways to functional outcomes in schizophrenia spectrum disorders: meta-analysis of social cognitive and neurocognitive predictors. Neurosci Biobehav Rev. (2019) 105:212–9. doi: 10.1016/j.neubiorev.2019.07.020, 31415864

[41] Fett A Viechtbauer W Dominguez M Penn D Os J Krabbendam L. The relationship between neurocognition and social cognition with functional outcomes in schizophrenia: a meta-analysis. Neurosci Biobehav Rev. (2011) 35:573–88. doi: 10.1016/j.neubiorev.2010.07.001 20620163

[42] Fingerman K Ng Y Huo M Birditt K Charles S Zarit S. Functional limitations, social integration, and daily activities in late life. J Gerontol B Psychol Sci Soc Sci. (2021) 76:1937–47. doi: 10.1093/geronb/gbab014, 33460446 PMC8598990

[43] Imamura H Uchiyama E Akiyama M Kaneko I Takebayashi T Nishiwaki Y. Relationship of living arrangement with the decline in functional capacity in elderly people by gender: a longitudinal observational study. Environ Health Prev Med. (2020) 25:15. doi: 10.1186/s12199-020-00853-w, 32434465 PMC7240989

[44] Kim Y Shelley M Lee J. The role of technology accessibility in social connectedness and health-related quality of life for rural older adults. J Aging Health. (2025) 2025:801. doi: 10.1177/08982643251336801, 40249964

[45] Han M Tan X Lee R Lee J Mahendran R. Impact of social media on health-related outcomes among older adults in Singapore: qualitative study. JMIR Aging. (2021) 4:e23826. doi: 10.2196/23826, 33595437 PMC8294634

[46] Chan C Burton K Flower R. Facilitators and barriers of technology adoption and social connectedness among rural older adults: a qualitative study. Health Psychol Behav Med. (2024) 12:2398167. doi: 10.1080/21642850.2024.2398167, 39234572 PMC11373358

[47] Bertolazzi A Quaglia V Bongelli R. Barriers and facilitators to health technology adoption by older adults with chronic diseases: an integrative systematic review. BMC Public Health. (2024) 24:506. doi: 10.1186/s12889-024-18036-5, 38365698 PMC10873991

